# Supplementary material for: Contribution of health system governance in delivering primary health care services for universal health coverage: A scoping review
Source: PLoS One. 2025 Feb 28;20(2):e0318244. doi: 10.1371/journal.pone.0318244 (PMC11870385; doi:10.1371/journal.pone.0318244)
Supplement: S2 Table — (DOCX) [file pone.0318244.s002.docx]

**Supplementary Information, Table S2: Tailored Search Terms for Different Databases.**

| **Database** | **Search terms** |
| --- | --- |
| **PubMed** | A: leadership[Title/Abstract] OR governance[Title/Abstract] OR stewardship[Title/Abstract] OR accountability[Title/Abstract] OR management[Title/Abstract] OR coordination[Title/Abstract] OR collaboration[Title/Abstract] OR regulation[Title/Abstract] OR multisectoral*[Title/Abstract] OR intersectoral*[Title/Abstract]  B: "Primary Health care"[Title/Abstract]  C: "universal health care"[Title/Abstract] OR "health services accessibility"[Title/Abstract] OR "quality of health care"[Title/Abstract] OR "safe health care"[Title/Abstract] OR "health coverage""[Title/Abstract] OR "health care coverage"[Title/Abstract] OR "health service coverage"[Title/Abstract] OR "universal coverage"[Title/Abstract] OR "universal health coverage"[Title/Abstract] OR UHC[Title/Abstract] OR "essential health coverage"[Title/Abstract] OR "health insurance coverage"[Title/Abstract] OR "financial risk protection"[Title/Abstract] OR "financial hardship"[Title/Abstract] OR "financial protection"[Title/Abstract] OR efficiency[Title/Abstract] OR equity[Title/Abstract] OR responsiveness[Title/Abstract] OR effectiveness[Title/Abstract] OR performance[Title/Abstract]  D: A AND B AND C |
| Web of science | **leadership OR governance OR stewardship OR accountability OR management OR coordination OR collaboration OR regulation OR multisectoral* OR intersectoral* (Abstract) and “Primary Health care” (Abstract) and “universal health care" OR “health services accessibility" OR "quality of health care" OR “safe health care” OR "health coverage", "health care coverage" OR "health service coverage" OR "universal coverage" OR "universal health coverage" OR UHC OR "essential health coverage" OR "health insurance coverage" OR "financial risk protection" OR "financial hardship" OR "financial protection" OR efficiency OR equity OR responsiveness OR effectiveness OR performance (Abstract)** |
| Scopus | ( ABS ( leadership  OR  governance  OR  stewardship  OR  accountability  OR  management  OR  coordination  OR  collaboration  OR  regulation  OR  multisectoral*  OR  intersectoral* )  AND  ABS ( "Primary Health care" )  AND  ABS ( "universal health care"  OR  "health services accessibility"  OR  "quality of health care"  OR  "safe health care"  OR  "health coverage" OR  "health care coverage"  OR  "health service coverage"  OR  "universal coverage"  OR  "universal health coverage"  OR  uhc  OR  "essential health coverage"  OR  "health insurance coverage"  OR  "financial risk protection"  OR  "financial hardship"  OR  "financial protection"  OR  efficiency  OR  equity  OR  responsiveness  OR  effectiveness  OR  performance ) ) |
| Cochrane | leadership OR governance OR stewardship OR accountability OR management OR coordination OR collaboration OR regulation OR multisectoral* OR intersectoral* in Title Abstract Keyword AND “Primary Health care” in Title Abstract Keyword AND “universal health care" OR “health services accessibility" OR "quality of health care" OR “safe health care” OR "health coverage" OR "health care coverage" OR "health service coverage" OR "universal coverage" OR "universal health coverage" OR UHC OR "essential health coverage" OR "health insurance coverage" OR "financial risk protection" OR "financial hardship" OR "financial protection" OR efficiency OR equity OR responsiveness OR effectiveness OR performance in Title Abstract Keyword |
| Embase | (leadership OR governance OR stewardship OR accountability OR management OR coordination OR collaboration OR regulation OR multisectoral* OR intersectoral*)/br AND ((“Primary Health care”):ab,ti) AND ((“universal health care" OR “health services accessibility" OR "quality of health care" OR “safe health care” OR "health coverage" OR "health care coverage" OR "health service coverage" OR "universal coverage" OR "universal health coverage" OR UHC OR "essential health coverage" OR "health insurance coverage" OR "financial risk protection" OR "financial hardship" OR "financial protection" OR efficiency OR equity OR responsiveness OR effectiveness OR performance):ab,ti) |
| PsycINFO | AB ( leadership OR governance OR stewardship OR accountability OR management OR coordination OR collaboration OR regulation OR multisectoral* OR intersectoral* ) AND AB “Primary Health care” AND AB ( “universal health care" OR “health services accessibility" OR "quality of health care" OR “safe health care” OR "health coverage" OR "health care coverage" OR "health service coverage" OR "universal coverage" OR "universal health coverage" OR UHC OR "essential health coverage" OR "health insurance coverage" OR "financial risk protection" OR "financial hardship" OR "financial protection" OR efficiency OR equity OR responsiveness OR effectiveness OR performance ) |
| Google scholar | A: leadership , governance stewardship, accountability, management, coordination, collaboration, regulation, multisectoral* , intersectoral*  B: “Primary Health care”  C: “universal health care", “health services accessibility", "quality of health care", “safe health care”, "health coverage", "health care coverage", "health service coverage" "universal coverage" "universal health coverage" , UHC, "essential health coverage", "health insurance coverage", "financial risk protection", "financial hardship" , "financial protection" , efficiency, equity, responsiveness, effectiveness, performance |
