# Supplementary material for: Contribution of health system governance in delivering primary health care services for universal health coverage: A scoping review
Source: PLoS One. 2025 Feb 28;20(2):e0318244. doi: 10.1371/journal.pone.0318244 (PMC11870385; doi:10.1371/journal.pone.0318244)
Supplement: S3 Table — (DOCX) [file pone.0318244.s003.docx]

**Supplementary Information, Table S3: Data Extraction on Health System Governance (HSG) in Primary Health Care (PHC) from Studies Included in the Review.**

| **Study** | **Country** | **type** | **Aim** | **Governance actions** | **Key findings** |
| --- | --- | --- | --- | --- | --- |
| Bedregal et al. | Chile | Qualitative | To assess the policy environment using stakeholder analysis | Evidence based management, human resources knowledge and skills for governance | - Management is not evidence based. - Culture and system organisation influence implementation - Appropriate skills of HR key for addresses the challenges. |
| Bell et al. | Namibia | Mixed methods | To draw lessons from a review of primary health care services | Regional and provincial management team | - Regional and provincial health management team are effective to address the needs of population and equitable PHC service delivery. |
| Frenk et al. | Mexico | review | To review of health policy review in Mexico | Health sector reform, primary health care model, horizontal integration | - Three generation of health service reform. - horizontal coordination in third generation, stability of leadership in the health sector, social protection in health for all. |
| Atun et al | Estonia | Mixed methods | To evaluate the health reforms | Multifaceted coordinated approach to PHC reforms | - New organisational structure, payment methods, training and services contracts, evidence-based guideline. - Impact- reduced hospitalisation of chronic conditions - Strong leadership and coordination - changes in laws; organisational restructuring; modifications to financing and provider payment systems; creation of incentives to enhance service innovations; investment in human resource development |
| Hunt et al | Multiple | Qualitative | To identifies some of the key right-to-health features of a health system | Right to health feature of health system | - building blocks:" leadership, governance, and stewardship. - the right to the highest attainable standard of health is not optional--and, like ethics, it recurs throughout all other approaches - transparency, participation, equity, and equality) such as transparency, participation, equity and equality, a comprehensive national health plan |
| Wakerman et al | Australia | Qualitative | To describe the factors and processes that facilitate or inhibit implementation, sustainability, | Environmental enablers- supportive policy; federal and state/territory relations; and community readiness. | - These enablers and services requirement improves effectiveness and lessens the threat to service sustainability. - five essential service requirements - governance, management, and leadership; funding; linkages; infrastructure; and workforce supply |
| Albin et al | Sweden and China | review | To describe and compare health care in Sweden and China | Health care systems- legislation, organisation, and finance | - Sweden there is one national law concerning health care. - China health under development law. - China has dominant hospital-based care in cities |
| Labonté | Not specified | Qualitative | To address several issues pertinent to health systems governance for health equity | Importance of two elements of good governance (transparency and participation) in health systems decision-making | - progressive health system reform and the implications of equity-focused health system governance on health workers' roles (identity as citizens). - political nature of health reforms based on equity; confrontation with power relations politics; managing efficiency and result based financing and equity and citizen empowerment. |
| Brixi et al | China | Mixed methods | To explore to what extent sub-national governments are addressing health inequities in China | Subnational expenditure for improved equity | - Regressive health expenditure at provincial level and are not aligned with national priorities. - Improve governance and financing public services delivery. - Weaknesses in local governance and capacity with national policies. - consolidate key health financing responsibilities at the provincial level and strengthen the accountability define targets for expenditure on PHC, outputs for each province use independent sources to monitor and evaluate policy implementation. |
| Cleary et al | LMICs | review | To review empirical literature about accountability mechanisms in LMICs | bureaucratic accountability | - Relatively power managers expectation make difficulty to respond to citizens. - supervision and management systems focused on compliance can constrain population priorities. |
| Nicholson et al | Multiple- UK, US, Canada, Australia | review | To synthesise the existing published literature on elements of current integrated primary/secondary health care | Integrated PHC governance | - joint planning; integrated information communication technology; change management; shared clinical priorities; incentives; population focus; measurement - using data as a quality improvement tool; continuing professional development supporting joint working; patient/community engagement; and innovation |
| Tulenko et al | Not specified | Qualitative | Perspective | Challenges of CHW programs | - Poor coordination and fragmentation, competing priorities, lack of support. - Integration with national system, career development opportunities, maintaining flexibility for programs and responds to local needs. |
| Veronesi et al | Australia | Qualitative | To provide a policy analysis of the Australian government's National Health Reform Agreement | Responsibilities of commonwealth and state/territories | - Commitment towards a funding arrangement based on uniform measures of performance. - a more decentralised nation-wide homogenisation of governance arrangements |
| Gonani et al | LMICs | Qualitative | To explore the leadership for PHC in LMICs in health-related documents. | Political and technical leadership | - inadequate political and technical leadership has prevented the realization of UHC in LIMCs in the last 30 years. |
| Nachtnebel et al | Asia pacific region | review | To explore on purchasing PHC services from the private sector | Coordination with private sector in service delivery | - Asia Pacific found positive effects with regards to access and utilisation of health services - vouchers and contracting influenced by contextual factors, appropriate selection of mechanism for services provided, and governance arrangements and stewardship capacity. |
| Abimbola et al et al | Nigeria | review | To understand how and under what circumstances the committee’s function, | Community health committee and models of governance | - Modes of functioning of committees- meeting, reaching committee, lobbying, inducing government support, taking control of health of community. - Power asymmetry of committee. - National guidelines are not clear on their accountability functions and minimum standards. |
| Foster et al | *Australia* | Qualitative | To examine how the various actors envisaged performance and the likely tensions surrounding population | Regional public health organisations and PHCOs | - the different rationalities at play and how different actors seek control of the policy space. - The political precariousness of PHCOs, and the wider difficulty of steering market-based professionals in the achievement of population health objectives. |
| Kress et al | Nigeria | Quantitative | To examine Nigeria's PHC system and possible causes of underperformance. | Performance of health system | - two overarching system-level challenges-financing and governance-that are key root causes of the dysfunctions observed in the PHC system-segmented supply chains; a lack of financial access to PHC; a lack of infrastructure, drugs, equipment, and vaccines at the facility level; and poor health worker performance. |
| Mugisha et al | Uganda | Qualitative | To identify governance related factors that promote/or hinder integration of mental health into PHC. | Mental health governance | - increased responsiveness/participation of key stakeholders. - Human resources, finances, medicines, and technologies constitute a major drawback to the integration of mental health into PHC. - Poor functioning at district level |
| Gurung et al | Nepal | Qualitative | To address this gap by reporting on research into complaint systems in primary health care (PHC) settings. | Health facility management committee | - HFMC members made verbal complaints. - No formal channel of complaints. - Reasons- power asymmetry, poor knowledge, perceived lack of responsiveness. |
| Lodenstein et al | LMICs | Review | To review cases of collective citizen action and advocacy with the aim to identify key mechanisms of provider responsiveness | Citizen’s engagement | - HW responsiveness- health providers' perceptions of the legitimacy of citizen groups, and citizen groups provide personal and professional support to health providers. - Citizens activated political or formal bureaucratic accountability channels - for health provider responsiveness comprise socio-political contexts in which providers self-identify as activists, health system contexts. |
| Petersen et al | LMICs – Africa and Asia | Qualitative | To identify key governance challenges, needs and potential strategies for integration of mental health into PHCs | Mental health governance | - strengthening capacity of subnational level to develop and implement integrated plans and promote responsiveness, intersectoral collaboration and community engagement. - Challenge- inadequate financing |
| Schneider et al | South Africa | Quantitative | To explore the leadership and governance tasks of large-scale CHW programmes at sub-national level | Roles of national CHWs program | - National mandates and subnational strategies of community-based services. - Organizational accountability for CHWs and stakeholders. - Integrated planning of HR finance. - leading change by building collective visions. |
| Tenbensel et al | Denmark and New Zealand | Review | To compare Denmark and New Zealand, two small countries with tax-funded health systems | Approach of health system. Incentives and intermediate organisations | - Denmark – soft hierarchy intrinsic motivation to improve quality, and - NZ- explicit hierarchical targets and financial incentives. |
| Upadhaya et al | Nepal | Qualitative | To appraise mental health systems governance in Nepal | Mental health governance in Nepal.  Public and not for profit sector. | - Enablers: Policy readiness, presence of NGOs and services users in policy forms and collaboration in implementation of mental health projects., - Barriers- failure in revision of policy and laws, lack of mental health act and regulations, lack of mental health units in ministry and district level, lack of training and drugs for services. |
| Assan et al | Ghana | Qualitative | to present the barriers to and facilitators of the CHPS | Community health planning scheme | - CHPS contribution particularly in bridging geographical access to health. - Limiting factors- governance and nuancing. - Addressing challenges need leadership to improve resources. |
| Campbell et al | Australia | Qualitative | To collate and analyse the contribution of ACCHSs | Aboriginal community-controlled health services | - ACCHSs improvement health through community-controlled governance, strengthening broader system and accessible CPHC provision. |
| Foster et al | Zambia | Qualitative | To build leadership and management competencies of rural facility heads, including increasing their ability to lead frontline teams | certificate in leadership and management practice program | - CLMP as continuous professional development program. - Strengthened leadership and management and ability to lead frontline workers. - Strengthened use of technology. |
| Gurung et al | Nepal | Qualitative | To describe community representation in Nepal's Health Facility Operation and Management Committees | Health facility management committees and degree of participation | - Participation of marginalised people in the committees. - Power elites influenced selection of committees. - Participation happened at manipulation and informing stage (Arnstein’ ladder) |
| Joulaei et al | MICs | Review | To present a very brief situational analysis of the health policy making and its outcomes in the MICs | Health policy making processes | - Challenges of governance and health system bb. - Policy making requires informed policy making, preparation of plan on sustainability and participation, focus on quality CPHC, reducing corruption and involvement of private sector/ technology, regulation, and health in all policies. |
| Lavoie et al | Canada | Mixed methods | To provide an analysis of the factors that shape PHC | Role of community health clinics in improving equity | - Three mechanisms contributed to equity: accountability, funding allocations, emergent priorities |
| Mabuchi et al | Nigeria | Qualitative | To understand the performance of PHCCs | Performance of PHCCs | - Areas of performance: community engagement, and staff management. Factors: contextual factors of providers, health center management, support from community leaders. Good center management can overcome these barriers. |
| S et al | Malaysia, Indonesia, Nigeria, and Australia | Review | To analyse leadership styles and functions of different countries in achieving leadership reform. | Leadership style in PHC program | - Democratic leadership in PHC system. - Participation of stakeholders and negotiation for resource allocation. - Challenges; government domination and lack of decentralisation. - Strategies: reform in leadership, alignment of top-down and bottom-up interventions |
| Tabrizi et al | Iran | qualitative | To identify the elements and infrastructures suitable for implementing NPM in the Iranian health complex. | New public management (decentralisation, market mechanism) | - Element of NPM- managerialism, decentralization, using market mechanism, performance management, customer orientation and performance budgeting. Implemented reform using inputs at individual level, system, and policy level. |
| Ward et al | Australia | Mixed methods | To determine the contextual influences associated with access arrangements | PHC model and local hospital network | - Supply of GP and financial viability influences on after-hours services. - Perceived need, referral networks influenced the provision of regular services. |
| Workie et al | Seychelles, including | Review | Review of experiences in the Seychelles. | High political commitment and downward accountability culture | - Public health system of the Seychelles succeeds due to high political commitment, strong voice and a downward accountability culture and investment in PH. - the epidemiological and demographic transitions. |
| Chu et al | Western pacific region | Qualitative | To provide an overview of health financing reforms across countries in the Western Pacific Region toward UHC | Health financing reforms and governance | - Health financing reforms improved services delivery and budget reforms. - Challenges- equity and efficiency and strengthening domestic financing. - Asian countries- moved to prepaid financing and government subsidy while the Pacific Island countries rely on government financing. |
| Dehnavieh et al | Iran | Qualitative | To identify the future trends of the primary health care in Iran | PHC and changing paradigm and emerging priority | - Changing paradigm from the volume to value in health services. - Emerging public health priority. - Internal and external factors of health system. |
| El-Jardali et al | LMICs | Review | To review the evidence on barriers and facilitators to the implementation of essential health benefit packages within PHC settings in LMICs | Involvement of different stakeholders | - Limited involvement of stakeholders and insufficient network and coordination. Facilitators: policy readiness. - Financial level: inadequate remunerations and facilitators- commitment to financing and new fundings. - Delivery level- inadequate delivery infrastructure while facilitators were defined package and training of providers. |
| Espinosa-Gonzalez et al | Europe | Qualitative | To analyse the interactions between PHC functions and their impact in PHC delivery | Correlation of decentralisation and governance and regulation | - health system governance determines the type of PHC financing. - the degree of decentralisation (both delegation and devolution) of PHC financing and regulation determines the governance functions. |
| avanparast et al | Australia | Mixed methods | To examine the strength and extent of collaborations between PHO and local government in population health planning. | Collaboration and partnership public health organisations and local governments | - Local health organisations (MLs and PHNs) reported limited time and financial support for collaboration with local government. - Organisational capacity and resources, supportive governance and public health legislation mandating a role for local governments were critical to collaborative planning |
| Serrate | Cuba | Qualitative | To explore the reflection of intersectorality in national regulations | Intersectionality in high level | - High-level political commitment promotes intersectorality as a strategy and encourage participation. Intersectorality increased the health system capacity. |
| Tabrizi et al | Iran | Qualitative | To obtain health professionals' perspectives about the suitable pillars and components of Iran's PHC | PHC governance and quality improvement | - Essential pillars of Iranian PHC governance model: quality improvement, management and leadership, community involvement and customer participation, effectiveness of PHC, human resource development, safety, health care evaluation and audit, and health information management |
| Yip et al | China | Review | To review the PHC reforms in China | Phase wise PHC reforms.  Systematic reforms. | - reforming its health-care delivery system- reforms of hospitals, and delivery system. China has made substantial progress in improving equal access to care Gaps in quality of care and NCDs, efficiency, expenditure, and public satisfaction. |
| Asmri et al | Saudi Arabia | review | To identify challenges within the Saudi Arabian health care system with a focus on PHC services. | Key areas for improvement in PHC system | - Areas include: the scope, structure, infrastructure, financing, increased demand, increased costs, and workforce capacity. - Challenges- inequity, quality, lack of information system, management and leadership and referral |
| Assefa et al | Ethiopia | review | To review of contribution of PHC | Interlinkage of PHC and UHC | - Policies, strategies, and programs are aligned with country priorities. - Diagonal approach to diseases control and system strengthening, community empowerment and multisectoral action that contributed to increase coverage |
| Carrillo et al | Salvador | Qualitative | To explore stakeholders' perceptions related to the management of NCDs in PHC | Intersectoral action of Salvadoran PHC system. | - Community engagement and the National Health Forum are ensuring accountability. - Challenges- coordination between levels of care and promotion program for NCDs. |
| Desta et al | Ethiopia | Quantitative | To compare district level capacity and performances between leadership, management, and governance | Leadership, management, and governance | - District level leadership development program contributes to improving district capacity, structure and management practices, and quality of care |
| Hassell et al | Caribbean region | Qualitative | To explain the government responsibility for reducing health inequities. | Accountability mechanism for equity and civic engagement | - Civil society has been recognized as a key partner in advancing sustainable and equitable national development. - The Healthy Caribbean Coalition has played a significant role in holding governments accountable for advancing health equity. |
| Langlois et al | LMICs | Qualitative | To analyse the primary health-care systems in 20 LMICs using a semi-grounded approach. | Funding and decentralisation, fragmentation for non-communicable diseases | - underfunding, corruption and poor engagement of informal workers, fragmentation of public and private systems hampered health insurance schemes. Poor regulation of private sector. - decentralization led to the fragmentation of PHC. - Regulation linked with financial incentives improved performance |
| Malakoane et al | South Africa | Mixed methods | To investigate providers perspectives of the health system challenges | Challenges of services delivery | - Challenges of PHC delivery- fragmentation, staff shortages, financial/cash-flow problems, risk to patient care, dysfunctional communication technology, 'poor information management. |
| Tumusiime et al | WHO African Region | Qualitative | To provide recommendations for continuity of services during crises | Health governance during crisis. | - Health services in the crisis- working multisectorally, moving from fragmentation to integration, ensuring implementation and knowledge exchange, rethinking resilience, and embracing antifragility |
| Abd Rahim et al | Sub-Saharan Africa and South Asia Region | Review | To synthesize the findings of empirical studies on the governance of integrated primary mental health care | Mental health care governance | - Challenges included lack of leadership and mental health prioritization, inadequate financing, and human resource capacity. Long terms system performance can address these challenges. - Integration of mental health in PHC is needed. |
| Chukwuma et al | Armenia | Mixed methods | To examine how purchasing has affected access to quality care in Armenia and its implications for the future reforms. | Health financing and governance | - Political priority of access to quality health care. - Decision making of purchaser without political interference. - the regulatory framework to ensure that revisions of the benefits package. regulations governing quality-related criteria for provider selection. payment incentives to increase the supply PHC services. increased pre-paid and pooled funds and better governance for quality services. |
| Clarke et al | Not specified | reviews | To strengthen PHC policymakers need guidance on how to allocate resources | Investment in PHC and telemedicine | - health workers' task shifting and telemedicine, can have positive economic impacts. - Gaps remain in the economic implications of models of PHC care |
| Dawa et al | India | Qualitative | To manage health at the district level and design framework for implementation | District as administrative unit for programme implementation | - district has a key role to play in implementing national programmes and in delivery of basic health service. - better management of existing resources and engaging stakeholders contribute to national health goals. Planning and managing health problems need an improved and responsive health governance. |
| Edelman et al | Not specified | review | To synthesize the current state of knowledge about PHC impacts, implementation enablers and barriers, and knowledge gaps across the three main PHC components | Implementation of PHC within the contexts | - investments in PHC improve equity and access, healthcare performance, accountability of health systems and health outcomes. - Enablers: equity-informed financing models, health system and governance frameworks. - Critical knowledge gaps remain in context-specific governance, financing, workforce, accountability, and service coordination mechanisms. |
| McCalman et al | Australia | Qualitative | To identify the barriers and enablers to transitioning the delivery of PHC services | PHC barriers and enablers at the organisational level | - Organisational level: internal lack of experience and capacity, lack of resources, transparency, - Enablers: leadership stability and capacity, community mandate, relationships with partner organisations |
| Simen-Kapeu et al | Liberia | Qualitative | To review the community health policy development process to draw lessons from the health system strengthening. | Health services and governance in the epidemic | - the importance of establishing a coordination mechanism and leveraging partnership support; using a systems approach to better inform policy shifts; strengthening community engagement; and conducting evidence-based planning to inform policymakers |
| Simen-Kapeu et al | Africa | Review | To identify common community health system bottlenecks | Bottlenecks for community health services and governance | - Challenges- health financing, essential medical technology, integrated services and community ownership and partnerships |
| Sitienei et al | Kenya | Qualitative | To examine the implementation of community participation, through collaborative governance in primary health care facilities | Health facility management committee,  Collaborative community engagement | - Poorly community engagement. - Poor focus on day to day functioning of the facility. - Community members of political connections or were retired government workers. - No formal mechanisms for stakeholder forum. - Poor clarity on collaborative governance |
| Sturmberg et al | Not specified | Qualitative | To review the WHO emphasis on UHC can only be achieved through PHC | Interlinkage of PHC and UHC, and funding | - Three pillars of PHC-enabling primary care and public health to integrate health services, empowering people, and communities to create healthy living conditions, and integrating multisectoral policy decisions. - Governments and/or funders at the top‐level not only promote the goals of the system (policies) but also constrain the system (rules, regulations, resources) in its ability to deliver. |
| Warren et al | Not specified | Qualitative | Opinion | System approach and community health system. | - the communities' roles and their interactions with other health system actors to accelerate outcomes and reflect the diversity of the community health ecosystem. cross-cutting priorities emerge-coverage, community health financing, policy change, institutionalization, resilience, accountability, community engagement, and whole-of-society efforts |
| Zaadoud et al | Multiple | review | To analyse and compare the frameworks of performance measurement in primary health care in the world. | performance in primary health care facilities | - a blatant need for performance measurement tools, relevant information, coherence between the operational and strategic levels, integration of organizational objectives in the measurement of performance to direct the structures towards a true management by quality |
| Adhikari et al | Nepal | review | To explore how the primary health care system is grappling the challenges amidst these changes | Renewed focus of PHC system. | - Transforming Nepal's primary health care system requires a clear focus on following priority areas: revised efforts towards strengthening of community based primary health care units; adapting vertical programs to federal governance; reinforcing the health insurance scheme; and strengthening an existing network of community health workers and health human resources. |
| Madon et al | India | Qualitative | To improve understanding of how the government's policy vision and instrumentation translate to interactions | Community health governance and coordination | - Three local governance mechanisms of horizontal coordination, demand for accountability and self-help help to explain improvements that have taken place at village level and contribute to the creation of a new theory of community health governance as evolving phenomena that requires a constant process of learning from the field to strengthen policymaking |
| Negash et al | Ethiopia | quantitative | To fill this research gap by assessing health system responsiveness and associated factors among outpatients from primary health care facilities | Health system responsiveness | - health system responsiveness performance was higher. - Confidentiality and dignity were the highest responsive domains. - To improve the health systems responsiveness and potentially fulfil patients' legitimate expectations, we need to facilitate informed treatment choice, provide reasonable care within a reasonable time frame, and give patients the option of consulting a specialist. |
| Kim et al | Uganda | Quantitative | To understand health facility management within the decentralized PHC system | Better facility management was associated with better essential drug availability | - Facilities with better management scores had better performance on several experiential quality measures. There were significant disparities in the management performance of PHC facilities including patients with greater wealth and education and those living in urban areas sought care at facilities that performed better on management. Private facilities and hospitals performed better on the management index |
| Klymchuk et al | Ukraine | Qualitative | to explore how conflict-affected communities in can develop sustainable mental health services in decentralised settings. | Connecting decentralization local stakeholders’ engagement, | - Decentralisation led to allocating funds alongside responsibilities for developing the services to communities. - Challenges were leadership, coordination, and collaboration problems; infrastructure, physical accessibility, and financial problems; PHC workforce shortage and lack of competencies; low awareness of available services and high stigma; war. - Communities stakeholders foresaw seven domains of action for decentralisation: increasing the role of communities and service users in the initiatives of governmental bodies; establishing in the communities local coordination/working groups dedicated to mental health service development; developing the community based spaces for integrated services provision; embedding the mental health services in the existing services; mental health advocacy and lobbying ladylike leaders and service users; increasing capacity of communities in financial management, fundraising; developing services by combining efforts and budgets of neighbouring communities |
| Lewis et al | Nepal | Qualitative | to identify best and worst performing primary health care centers in Nepal and investigated perceived drivers of best performance. | inform quality improvement efforts and health system reforms | - Best performers were effective management capacity, engaging local leadership, building active community accountability, assessing, and responding to facility performance, developing sources of funding, compensating staff fairly, managing clinical staff performance, and promoting uninterrupted availability of supplies and equipment |
| Pope et al | Mozambique | Quantitative | To describe the association between management effectiveness and facility readiness to provide FP services | Degree of management effectiveness associated with an increased improved FP service readiness. | - Facility management effectiveness, and urban location were significantly associated with higher levels of readiness for FP service delivery |
| Ruhago et al | Tanzania | Quantitative | To assess the functionality of the FFARS in management, accounting, and reporting funds received and disbursed in the stride forward strengthening public financial management in PHC facilities | Potential in improving facility financial management | - The user fee was the predominant source of revenue, in urban facilities, while revenue from health insurance was not among the top three highest sources of revenue. - Expenditure priorities leaned more towards drugs and supplies followed by allowances and training |
| Alemu et al | Ethiopia | Quantitative | To assesses factors affecting the inventory management performance | Performance of inventory management performance | - Inventory management performance decreases peripheral facilities. A positive correlation between the availability of supplies, stocks and reporting accuracy. The inventory accuracy was significantly different between primary hospitals and health posts; and health centers and health posts |
| Aranda et al | El Salvador | Qualitative | To explore the behavioural and contextual factors that may have contributed to the gains in performance. | system-wide effects of social interactions and relationships | - Increased social interactions and relationships among implementers that enhanced communication and created opportunities for social learning; cyclical performance monitoring that generated novel flows of information. - The social nature of implementation processes and describe plausible pathways through which lower-order implementation programme effects can contribute to higher-order changes in system performance |
| El-Shal et al | Egypt | Quantitative | To estimate the effects of discontinuing PBF in FP and MNCH outcomes. | Effectiveness of performance-based financing | - Discontinuing performance-based incentives to providers had a negative effect on the knowledge of contraceptive methods, iron supplementation during pregnancy, the prevalence of childhood acute respiratory infection, and, more importantly, under-five child mortality, targeted by the performance-based financing scheme. |
| Kesale et al | Tanzania | Quantitative | To assess the perceived performance of the HFGCs and the associated factors in overseeing the healthcare services delivery at the primary health facilities | Contribution of health facility governance committees | - The main factors associated with the perceived low performance of the health facility governing committee members were age, level of education and duration served in the committee. - poor perceived performance due to a low level of education, increasing age, serving as a HFGC member for less than 1 year. |
| Sambodo et al | Indonesia | Quantitative | To evaluate the effects of KBK on its three incentivized monthly outcomes | Performance-based capitation reform | - Positive effects of KBK on two out of three outcomes, all estimated effect sizes leave the actual rates far below the program targets |
