# Supplementary material for: Contribution of health system governance in delivering primary health care services for universal health coverage: A scoping review
Source: PLoS One. 2025 Feb 28;20(2):e0318244. doi: 10.1371/journal.pone.0318244 (PMC11870385; doi:10.1371/journal.pone.0318244)
Supplement: S4 Table — (DOCX) [file pone.0318244.s004.docx]

**Supplementary Information, Table S4: Descriptive Summary of Studies Included in the Review [1-74].**

| **Study** | **Country** | **Health system governance interventions** |
| --- | --- | --- |
| Bedregal et al. | Chile | Evidence-based management and human resources skills for HSG |
| Bell et al. | Namibia | Regional and provincial management team |
| Frenk et al. | Mexico | Health sector reform, PHC model, horizontal integration |
| Atun et al | Estonia | Multifaceted coordinated approach to PHC reforms |
| Hunt et al | Multi-country | Right to health feature of the health system |
| Wakerman et al | Australia | Environmental enablers- supportive policy; federal and state/territory relations; and community readiness. |
| Albin et al | Multi-country | Legislation, organisation, and finance in Sweden and China |
| Labonté | Not specified | elements of good governance (transparency and participation) and decision-making |
| Brixi et al | China | Subnational expenditure for improved equity |
| Cleary et al | LMICs | bureaucratic accountability |
| Nicholson et al | Multi-country | Integrated PHC governance in the UK, US, Canada, Australia |
| Tulenko et al | Not specified | Challenges of CHW programs |
| Veronesi et al | Australia | Responsibilities of commonwealth and state/territories |
| Gonani et al | LMICs | Political and technical leadership |
| Nachtnebel et al | Asia pacific | Coordination with the private sector in service delivery |
| Abimbola et al | Nigeria | Community health committee and models of governance |
| Foster et al | Australia | Regional public health organisations and PHCOs |
| Kress et al | Nigeria | Performance of health system |
| Mugisha et al | Uganda | Mental health governance |
| Gurung et al | Nepal | Health facility management committee |
| Lodenstein et al | LMICs | Citizen’s engagement |
| Petersen et al | Multi-country | Mental health governance in Africa and Asia |
| Schneider et al | South Africa | Roles of national CHWs program |
| Tenbensel et al | Multi-country | Approach of health system, and incentives and intermediate organisations in Denmark and New Zealand |
| Upadhaya et al | Nepal | Mental health governance, and public and not-for-profit sector. |
| Assan et al | Ghana | Community health planning scheme |
| Campbell et al | Australia | Aboriginal community-controlled health services |
| Foster et al | Zambia | certificate in leadership and management practice program |
| Gurung et al | Nepal | Health facility management committees and participation |
| Joulaei et al | MICs | Health policy-making processes |
| Lavoie et al | Canada | Role of community health clinics in improving equity |
| Mabuchi et al | Nigeria | Performance of PHCCs |
| Syafinaz S et al | Multi-country | Leadership style in PHC program in Malaysia, Indonesia, Nigeria, Australia |
| Tabrizi et al | Iran | New public management (decentralisation, market mechanism) |
| Ward et al | Australia | PHC model and local hospital network |
| Workie et al | Seychelles | High political commitment and downward accountability |
| Chu et al | Western pacific | Health financing reforms and governance |
| Dehnavieh et al | Iran | PHC and changing paradigm and emerging priority |
| El-Jardali et al | LMICs | Involvement of different stakeholders |
| Espinosa-Gonzalez et al | Europe | Correlation of decentralisation and governance and regulation |
| Javanparast et al | Australia | Partnerships with public health organisations and local governments |
| Serrate | Cuba | Intersectorality in high-level |
| Tabrizi et al | Iran | PHC governance and quality improvement |
| Yip et al | China | Phase wise PHC reforms, systematic reforms. |
| Asmri et al | Saudi Arabia | Key areas for improvement in PHC system |
| Assefa et al | Ethiopia | Interlinkage of PHC and UHC |
| Carrillo et al | Salvador | Intersectoral action of Salvadoran PHC system. |
| Desta et al | Ethiopia | Leadership, management, and governance |
| Hassell et al | Caribbean | Accountability mechanism for equity and civic engagement |
| Langlois et al | LMICs | Funding and decentralisation, fragmentation for NCDs |
| Malakoane et al | South Africa | Challenges of service delivery |
| Tumusiime et al | WHO Africa | Health governance during crisis. |
| Abd Rahim et al | Multi-country | Mental health governance in Sub-Saharan Africa and South Asia |
| Chukwuma et al | Armenia | Health financing and governance |
| Clarke et al | Not specified | Investment in PHC and telemedicine |
| Dawa et al | India | District as the administrative unit for programme implementation |
| Edelman et al | Not specified | Implementation of PHC within the contexts |
| McCalman et al | Australia | PHC barriers and enablers at the organisational level |
| Simen-Kapeu et al | Liberia | Health services and governance in the epidemic |
| Simen-Kapeu et al | Africa | Bottlenecks for community health services and governance |
| Sitienei et al | Kenya | Health facility management committee,  Collaborative community engagement |
| Sturmberg et al | Not specified | Interlinkage of PHC and UHC and funding |
| Warren et al | Not specified | System approach and community health system. |
| Zaadoud et al | Multi-country | performance in primary health care facilities |
| Adhikari et al | Nepal | Renewed focus of PHC system. |
| Madon et al | India | Community health governance and coordination |
| Negash et al | Ethiopia | Health system responsiveness |
| Kim et al | Uganda | Better facility management and availability of essential drug |
| Klymchuk et al | Ukraine | Connecting decentralization local stakeholders’ engagement |
| Lewis et al | Nepal | Inform quality improvement efforts and health system reforms |
| Pope et al | Mozambique | Degree of management effectiveness and service readiness. |
| Ruhago et al | Tanzania | Potential in improving facility financial management |
| Alemu et al | Ethiopia | Performance of inventory management performance |
| Aranda et al | El Salvador | System-wide effects of social interactions and relationships |
| El-Shal et al | Egypt | Effectiveness of performance-based financing |
| Kesale et al | Tanzania | Contribution of health facility governance committees |
| Sambodo et al | Indonesia | Performance-based capitation reform |

1. Abd Rahim A, Abdul Manaf R et al: Health System Governance for the Integration of Mental Health Services into Primary Health Care in the Sub-Saharan Africa and South Asia Region: A Systematic Review. *Inquiry* 2021, 58:469580211028579.

2. Abimbola S, Molemodile SK et al: 'The government cannot do it all alone': realist analysis of the minutes of community health committee meetings in Nigeria. *Health Policy Plan* 2016, 31(3):332-345.

3. Adhikari B, Mishra SR et al: Transforming Nepal's primary health care delivery system in global health era: addressing historical and current implementation challenges. *Global Health* 2022, 18(1):8.

4. Al Asmri M, Almalki MJ et al: The public health care system and primary care services in Saudi Arabia: a system in transition. *East Mediterr Health J* 2020, 26(4):468-476.

5. Albin B, Hjelm K et al: Health care systems in Sweden and China: Legal and formal organisational aspects. *Health Res Policy Syst* 2010, 8:20.

6. Alemu AA, Fenta TG et al: Factors Affecting Inventory Management Performance of Tracer Medicines Across Primary Health Care Units, Gamo Zone, Southern Nations Nationalities and People's Region, Ethiopia. *Integr Pharm Res Pract* 2023, 12:49-60.

7. Aranda LE, Arif Z et al: Characterizing the implementation of performance management interventions in a primary health care system: a case study of the Salud Mesoamerica Initiative in El Salvador. *Health Policy Plan* 2023, 38(5):579-592.

8. Assan A, Takian A et al: Universal health coverage necessitates a system approach: an analysis of Community-based Health Planning and Services (CHPS) initiative in Ghana. *Global Health* 2018, 14(1):107.

9. Assefa Y, Hill PS et al: Primary health care contributions to universal health coverage, Ethiopia. *Bull World Health Organ* 2020, 98(12):894-905A.

10. Atun RA, Menabde N et al: Introducing a complex health innovation--primary health care reforms in Estonia (multimethods evaluation). *Health Policy* 2006, 79(1):79-91.

11. Bedregal P, Ferlie E: Evidence based primary care? A multi-tier, multiple stakeholder perspective from Chile. *Int J Health Plann Manage* 2001, 16(1):47-60.

12. Bell R, Ithindi T et al: Improving equity in the provision of primary health care: lessons from decentralized planning and management in Namibia. *Bull World Health Organ* 2002, 80(8):675-681.

13. Brixi H, Mu Y et al: Engaging sub-national governments in addressing health equities: challenges and opportunities in China's health system reform. *Health Policy Plan* 2013, 28(8):809-824.

14. Campbell MA, Hunt J et al: Contribution of Aboriginal Community-Controlled Health Services to improving Aboriginal health: an evidence review. *Aust Health Rev* 2018, 42(2):218-226.

15. Chu A, Kwon S et al: Health Financing Reforms for Moving towards Universal Health Coverage in the Western Pacific Region. *Health Syst Reform* 2019, 5(1):32-47.

16. Clarke L, Anderson M et al: Economic Aspects of Delivering Primary Care Services: An Evidence Synthesis to Inform Policy and Research Priorities. *Milbank Q* 2021, 99(4):974-1023.

17. Cleary SM, Molyneux S et al: Resources, attitudes and culture: an understanding of the factors that influence the functioning of accountability mechanisms in primary health care settings. *BMC Health Serv Res* 2013, 13:320.

18. Dawa N, Narayan T et al: Managing Health at District Level: A Framework for Enhancing Programme Implementation in India. *Journal of Health Management* 2021, 23(1):119-128.

19. Desta BF, Abitew A et al: Leadership, governance and management for improving district capacity and performance: the case of USAID transform: primary health care. *BMC Fam Pract* 2020, 21(1):252.

20. Edelman A, Marten R et al: Modified scoping review of the enablers and barriers to implementing primary health care in the COVID-19 context. *Health Policy Plan* 2021, 36(7):1163-1186.

21. El-Jardali F, Fadlallah R et al: Barriers and facilitators to implementation of essential health benefits package within primary health care settings in low-income and middle-income countries: A systematic review. *Int J Health Plann Manage* 2019, 34(1):15-41.

22. El-Shal A, Cubi-Molla P et al: Discontinuation of performance-based financing in primary health care: impact on family planning and maternal and child health. *Int J Health Econ Manag* 2023, 23(1):109-132.

23. Espinosa-Gonzalez AB, Delaney BC et al: The impact of governance in primary health care delivery: a systems thinking approach with a European panel. *Health Res Policy Syst* 2019, 17(1):65.

24. Foster AA, Makukula MK et al: Strengthening and Institutionalizing the Leadership and Management Role of Frontline Nurses to Advance Universal Health Coverage in Zambia. *Glob Health Sci Pract* 2018, 6(4):736-746.

25. Foster M, Henman P et al: Population health performance as primary healthcare governance in Australia: professionals and the politics of performance. *Policy Studies* 2016, 37(6):521-534.

26. Frenk J, Sepulveda J et al: Evidence-based health policy: three generations of reform in Mexico. *Lancet* 2003, 362(9396):1667-1671.

27. Gonani A, Muula AS: The importance of Leadership towards universal health coverage in Low Income Countries. *Malawi Med J* 2015, 27(1):34-37.

28. Gurung G, Derrett S et al: Why service users do not complain or have 'voice': a mixed-methods study from Nepal's rural primary health care system. *BMC Health Serv Res* 2017, 17(1):81.

29. Gurung G, Derrett S et al: Nepal's Health Facility Operation and Management Committees: exploring community participation and influence in the Dang district's primary care clinics. *Prim Health Care Res Dev* 2018, 19(5):492-502.

30. Hassell TA, Hutton MT et al: Civil society promoting government accountability for health equity in the Caribbean: The Healthy Caribbean Coalition. *Rev Panam Salud Publica* 2020, 44:e79.

31. Hunt P, Backman G: Health systems and the right to the highest attainable standard of health. *Health Hum Rights* 2008, 10(1):81-92.

32. Javanparast S, Baum F et al: Collaborative population health planning between Australian primary health care organisations and local government: lost opportunity. *Aust N Z J Public Health* 2019, 43(1):68-74.

33. Jimenez Carrillo M, Leon Garcia M et al: Comprehensive primary health care and non-communicable diseases management: a case study of El Salvador. *Int J Equity Health* 2020, 19(1):50.

34. Joulaei H, Heydari M: Health Policy-Making Requirements to Attain Universal Health Coverage in the Middle-Income Countries: A Brief Report. *Shiraz E-Medical Journal* 2018, In Press(In Press).

35. Kesale AM, Jiyenze MK et al: Perceived performance of health facility governing committees in overseeing healthcare services delivery in primary health care facilities in Tanzania. *Int J Health Plann Manage* 2023, 38(1):239-251.

36. Kim JH, Bell GA et al: Health facility management and primary health care performance in Uganda. *BMC Health Serv Res* 2022, 22(1):275.

37. Klymchuk V, Vysotska K et al: Decentralisation and community stakeholders’ engagement for better mental health services development in the conflict-affected regions of Ukraine. *Journal of Public Mental Health* 2022, 21(4):288-302.

38. Kress DH, Su Y et al: Assessment of Primary Health Care System Performance in Nigeria: Using the Primary Health Care Performance Indicator Conceptual Framework. *Health Syst Reform* 2016, 2(4):302-318.

39. Labonte R: Health Systems Governance for health equity: critical reflections. *Rev Salud Publica (Bogota)* 2010, 12 Suppl 1:62-76.

40. Langlois EV, McKenzie A et al: Measures to strengthen primary health-care systems in low- and middle-income countries. *Bull World Health Organ* 2020, 98(11):781-791.

41. Lavoie JG, Varcoe C et al: Sentinels of inequity: examining policy requirements for equity-oriented primary healthcare. *BMC Health Serv Res* 2018, 18(1):705.

42. Lewis TP, Aryal A et al: Best and worst performing health facilities: A positive deviance analysis of perceived drivers of primary care performance in Nepal. *Soc Sci Med* 2022, 309:115251.

43. Lodenstein E, Dieleman M et al: Health provider responsiveness to social accountability initiatives in low- and middle-income countries: a realist review. *Health Policy Plan* 2017, 32(1):125-140.

44. Mabuchi S, Sesan T et al: Pathways to high and low performance: factors differentiating primary care facilities under performance-based financing in Nigeria. *Health Policy Plan* 2018, 33(1):41-58.

45. Madon S, Krishna S: Theorizing community health governance for strengthening primary healthcare in LMICs. *Health Policy Plan* 2022, 37(6):706-716.

46. Malakoane B, Heunis JC et al: Public health system challenges in the Free State, South Africa: a situation appraisal to inform health system strengthening. *BMC Health Serv Res* 2020, 20(1):58.

47. McCalman J, Jongen CS et al: The Barriers and Enablers of Primary Healthcare Service Transition From Government to Community Control in Yarrabah: A Grounded Theory Study. *Front Public Health* 2021, 9:616742.

48. Mugisha J, Ssebunnya J et al: Towards understanding governance issues in integration of mental health into primary health care in Uganda. *Int J Ment Health Syst* 2016, 10(1):25.

49. Nachtnebel M, O'Mahony A et al: Effectively engaging the private sector through vouchers and contracting - A case for analysing health governance and context. *Soc Sci Med* 2015, 145:193-200.

50. Negash WD, Tsehay CT et al: Health system responsiveness and associated factors among outpatients in primary health care facilities in Ethiopia. *BMC Health Serv Res* 2022, 22(1):249.

51. Nicholson C, Jackson C et al: A governance model for integrated primary/secondary care for the health-reforming first world - results of a systematic review. *BMC Health Serv Res* 2013, 13:528.

52. Petersen I, Marais D et al: Strengthening mental health system governance in six low- and middle-income countries in Africa and South Asia: challenges, needs and potential strategies. *Health Policy Plan* 2017, 32(5):699-709.

53. Pope S, Augusto O et al: Primary Health Care Management Effectiveness as a Driver of Family Planning Service Readiness: A Cross-Sectional Analysis in Central Mozambique. *Glob Health Sci Pract* 2022, 10(Suppl 1).

54. Ruhago GM, Ngalesoni FN et al: Strengthening financial management systems at primary health care: Performance assessment of the Facility Financial Accounting and Reporting System (FFARS) in Tanzania. *Front Health Serv* 2022, 2:787940.

55. S IS, Azreena E et al: Review of leadership function and reform towards enhancing primary health care services in selected countries. *International Journal of Public Health & Clinical Sciences (IJPHCS)* 2018, 5(1):36-52.

56. Sambodo NP, Bonfrer I et al: Effects of performance-based capitation payment on the use of public primary health care services in Indonesia. *Soc Sci Med* 2023, 327:115921.

57. Schneider H, Nxumalo N: Leadership and governance of community health worker programmes at scale: a cross case analysis of provincial implementation in South Africa. *Int J Equity Health* 2017, 16(1):72.

58. Serrate PCF: Universal Health in Cuba: Healthy Public Policy in All Sectors. *MEDICC REVIEW* 2019, 21(4):74-77.

59. Simen-Kapeu A, Lewycka S et al: Strengthening the community health program in Liberia: Lessons learned from a health system approach to inform program design and better prepare for future shocks. *J Glob Health* 2021, 11:07002.

60. Simen-Kapeu A, Reserva ME et al: Galvanizing Action on Primary Health Care: Analyzing Bottlenecks and Strategies to Strengthen Community Health Systems in West and Central Africa. *Glob Health Sci Pract* 2021, 9(Suppl 1):S47-S64.

61. Sitienei J, Manderson L et al: Community participation in the collaborative governance of primary health care facilities, Uasin Gishu County, Kenya. *PLoS One* 2021, 16(3):e0248914.

62. Sturmberg JP, Martin CM: Universal health care - A matter of design and agency? *J Eval Clin Pract* 2021, 27(5):1011-1017.

63. Tabrizi JS, HaghGoshayie E et al: New public management in Iran's health complex: a management framework for primary health care system. *Prim Health Care Res Dev* 2018, 19(3):264-276.

64. Tabrizi JS, Pourasghar F et al: Governance of Iranian Primary Health Care System: Perceptions of Experts. *Iran J Public Health* 2019, 48(3):541-548.

65. Tenbensel T, Burau V: Contrasting approaches to primary care performance governance in Denmark and New Zealand. *Health Policy* 2017, 121(8):853-861.

66. Tulenko K, Mogedal S et al: Community health workers for universal health-care coverage: from fragmentation to synergy. *Bull World Health Organ* 2013, 91(11):847-852.

67. Tumusiime P, Karamagi H et al: Building health system resilience in the context of primary health care revitalization for attainment of UHC: proceedings from the Fifth Health Sector Directors' Policy and Planning Meeting for the WHO African Region. *BMC Proc* 2020, 14(Suppl 19):16.

68. Upadhaya N, Jordans MJD et al: Current situations and future directions for mental health system governance in Nepal: findings from a qualitative study. *Int J Ment Health Syst* 2017, 11(1):37.

69. Veronesi G, Harley K et al: Governance, transparency and alignment in the Council of Australian Governments (COAG) 2011 National Health Reform Agreement. *Aust Health Rev* 2014, 38(3):288-294.

70. Wakerman J, Humphreys JS et al: Features of effective primary health care models in rural and remote Australia: a case-study analysis. *Med J Aust* 2009, 191(2):88-91.

71. Ward B, Lane R et al: Context matters for primary health care access: a multi-method comparative study of contextual influences on health service access arrangements across models of primary health care. *Int J Equity Health* 2018, 17(1):78.

72. Warren CE, Bellows B et al: Strength in Diversity: Integrating Community in Primary Health Care to Advance Universal Health Coverage. *Glob Health Sci Pract* 2021, 9(Suppl 1):S1-S5.

73. Workie NW, Shroff E et al: Who Needs Big Health Sector Reforms Anyway? Seychelles' Road to UHC Provides Lessons for Sub-Saharan Africa and Island Nations. *Health Syst Reform* 2018, 4(4):362-371.

74. Yip W, Fu H et al: 10 years of health-care reform in China: progress and gaps in Universal Health Coverage. *Lancet* 2019, 394(10204):1192-1204.
