# Supplementary material for: Contribution of health system governance in delivering primary health care services for universal health coverage: A scoping review
Source: PLoS One. 2025 Feb 28;20(2):e0318244. doi: 10.1371/journal.pone.0318244 (PMC11870385; doi:10.1371/journal.pone.0318244)
Supplement: Attachment — Submitted filename: Review Governance.doc [file pone.0318244.s005.doc]

- The authors need to address inconsistencies in the document, In the abstract, the authors write that they used 64 studies in the review, but in the main document there are 74 studies.

In addition, the purpose of this scoping review is to explore the function of HSG in the context of PHC settings towards UHC but in the methodology, the research questions focused on contributions of HSG to the delivery of PHC services. The purpose fails to capture UHC which is consistently positioned as very important in the topic of HSG and PHC.

- It is not clear what was done to the broad research questions, in addition it is not clear which is the broad research question and which is the final reviewed research question..see Lines 119-120
- Why were the six databases selected, a justification for this choice is essential.
- Line 116: The author should discuss the three concepts referred to here in this line.
- I suggest that authors avoid the use of disagreements by the reviewers on varying issues e.g Line 146…
- In Line 192, it is not clear how many of these studies were from, El Salvador, Ukraine, Egypt, Indonesia, and Kenya
- In line 499, digital tools cannot form policies,
- In lines 285-line 286 (who is empowered?)
- In lines 142-143. Were there specific interventions the authors were referring to?
- Line 87-98 the sentence is incomplete.
