# Supplementary material for: Contribution of health system governance in delivering primary health care services for universal health coverage: A scoping review
Source: PLoS One. 2025 Feb 28;20(2):e0318244. doi: 10.1371/journal.pone.0318244 (PMC11870385; doi:10.1371/journal.pone.0318244)
Supplement: Attachment — Submitted filename: PHG comments__.docx [file pone.0318244.s006.docx]

**Title:** The title needs to be reviewed. “Governance of primary health care system”. What is the gap in the primary health care system? Are you talking about the influence of governance? Are you talking about the impact of healthcare governance on primary health system? The title needs to be amended. The title and objective must be linked. “In the objective, you have “explores function” and “UHC”. The title should have UHC.

Kindly refer to your research question to formulate the title “Line 115” “We identified the research question focusing on the contribution of HSG to delivery of PHC services”

**1. Introduction**

1. The first paragraph of introduction is having some repetitions: Line52 and Line55:

Line52: Health system governance (HSG) is the rules (formal and informal) for collective action

Line55: Additionally, HSG is “as the collective actions…”

Line 59-63: This paragraph is not clear. Kindly break the paragraph in to pieces to make it easy to understand for the reader

There are inconsistencies between the aim given in abstract and the one given in the introduction.

Line25: In the abstract the aim of the study: “This scoping review explores the function of HSG in the context of PHC setting towards UHC”.

Line102: The aim of the study; “This study aimed to synthesize HSG interventions' contribution to delivering and utilizing PHC services”

Page64-82: this paragraph enumerates frameworks: when I read these frameworks, I would expect to see the frameworks utilized in the document but it is not the case.

Instead, kindly discuss at least 3 studies on health systems governance of primary healthcare in the introduction and let the 3 studies support the discussion and universal health coverage!

Introduction should also briefly discuss Health systems blocks because these must comeback in the discussion.

**2. Methodology**

This framework of Arksey and O'Malley should be briefly described

Line 116: We conceptualized three concepts: role of HSG to deliver health service to achieve UHC; Where are the 3 concepts????

**Identifying relevant studies**

1. What guided the choice of the 6 databases? I have a concern on why you have chosen “PsychInfo” as one of the databases to search from. PsychInfo is a database of abstracts and articles in the field of psychology and psychiatry. Why did you choose this database? This needs a strong justification.

2. For each database, provide the search strategy including the search terms. Provide also the search strategy for google scholar engine.

**Study selection**

It is not clear how you identified relevant studies. Kindly provide clearly inclusion and exclusion criteria

**Summarizing and reporting**

It is confusing how you summarized and reported the results. Kindly elaborate how you did a synthesis in details, how you grouped the papers according to the groups they belong

**3. Results**

PRISMA: “Excluded as did not match purpose of the review”. This statement is not simply enough, kindly elaborate for each paper the reason of exclusion.

Line183: “In the final review, we included 64 studies”, yet in the PRISMA, the number of papers are 74.

**Descriptive characteristics of included studies**

Kindly describe all papers identified by country, type of paper, author and year with citation of each paper

Move Table1 to supplementary documents

Line 202: Fig2 presents the summary of HSG interventions in the PHC context

Is this Fig2 part of your results? In which paper did you extract this fig2? If the information of figure2 is not among the results, kindly put it in the introduction or literature review

Line 164: You said that to summarize the findings you used WHO framework on governance and financing: Looking in to the summary, they are many subtitles which are not part of WHO framework such multi-sectoral engagement (line 438), community engagement (line 459),….

Line 570: Our purpose of the review was to synthesize evidence rather than grade the evidence, however, the results are a simple summary of what was reported in the studies, on the contrary, the paper should synthesize the data as opposed to summarize. Results should have a synthesis and not a summary.

In the aim of the study and methodology, Universal health coverage appeared both in aim and search but does not come out among findings. What happened in the findings? Is it that no studies reported universal coverage?

4. Discussion: Discuss how performing HSG (countries) are doing, what special strategies they have in terms of HSG which are making them performing and their strengths and weaknesses. Discuss countries in pools of LMICs and HICs, what are differences

**Conclusion**

The conclusion is not supporting the findings at all. Kindly relate your findings to the presented results
