# Supplementary material for: Contribution of health system governance in delivering primary health care services for universal health coverage: A scoping review
Source: PLoS One. 2025 Feb 28;20(2):e0318244. doi: 10.1371/journal.pone.0318244 (PMC11870385; doi:10.1371/journal.pone.0318244)
Supplement: Attachment — Submitted filename: Response to reviwers_R1.doc [file pone.0318244.s007.doc]

# Title: Contribution of health system governance on delivery of primary health care services towards universal health coverage: a scoping review

Manuscript ID: PONE-D-23-27336

Point by point to the editor's and reviewers’ comments

The authors’ team would like to thank you for your insightful and constructive feedback on our manuscript. We have revised it as suggested. In this document, we have responded point-by-point to your comments and clarified the concerns where necessary.

Additional Editor Comments:

| **Additional Editor Comments:** | **Response** |
| --- | --- |
| Title: The title needs to be reviewed. “Governance of primary health care system”. What is the gap in the primary health care system? Are you talking about the influence of governance? Are you talking about the impact of healthcare governance on primary health system? The title needs to be amended. The title and objective must be linked. “In the objective, you have “explores function” and “UHC”. The title should have UHC. Kindly refer to your research question to formulate the title “Line 115” “We identified the research question focusing on the contribution of HSG to delivery of PHC services” | We have revised the title to include universal health coverage. Our focus was to synthesize the contribution of governance to the delivery of PHC services towards UHC. The manuscript's focus has been revised to include a broader focus on the contribution of HSG in the delivery of PHC services towards UHC. |
| . Introduction |  |
| 1. The first paragraph of introduction is having some repetitions: Line52 and Line55: Line52: Health system governance (HSG) is the rules (formal and informal) for collective action Line55: Additionally, HSG is “as the collective actions…” Line 59-63: This paragraph is not clear. Kindly break the paragraph in to pieces to make it easy to understand for the reader | We have revised these as suggested. Repetitions were removed, and the paragraph is broken and made clearer. |
| There are inconsistencies between the aim given in abstract and the one given in the introduction. Line25: In the abstract the aim of the study: “This scoping review explores the function of HSG in the context of PHC setting towards UHC”. Line102: The aim of the study; “This study aimed to synthesize HSG interventions' contribution to delivering and utilizing PHC services” | These were revised and made consistent. We focused on HSG's contribution to delivering PHC services for UHC. |
| Page64-82: this paragraph enumerates frameworks: when I read these frameworks, I would expect to see the frameworks utilized in the document but it is not the case. Instead, kindly discuss at least 3 studies on health systems governance of primary healthcare in the introduction and let the 3 studies support the discussion and universal health coverage! | We thank the editor for this important insight. As suggested in the introduction and discussion section, we have included studies. |
| Introduction should also briefly discuss Health systems blocks because these must comeback in the discussion. | We revised it as suggested. We included WHO BB in both the introduction and discussion sections. |
| 2. Methodology |  |
| This framework of Arksey and O'Malley should be briefly described | We briefly described the framework and followed the steps suggested by the framework. |
| Line 116: We conceptualized three concepts: role of HSG to deliver health service to achieve UHC; Where are the 3 concepts???? | We mentioned these three concepts- HSG, PHC and UHC |
| Identifying relevant studies 1. What guided the choice of the 6 databases? I have a concern on why you have chosen “PsychInfo” as one of the databases to search from. PsychInfo is a database of abstracts and articles in the field of psychology and psychiatry. Why did you choose this database? This needs a strong justification. 2. For each database, provide the search strategy including the search terms. Provide also the search strategy for google scholar engine. | We described why these databases were included in the search, including using GS. Multiple databases were used to capture important and relevant studies on the topic. |
| Study selection It is not clear how you identified relevant studies. Kindly provide clearly inclusion and exclusion criteria | We included the inclusion and exclusion criteria. |
| Summarizing and reporting It is confusing how you summarized and reported the results. Kindly elaborate how you did a synthesis in details, how you grouped the papers according to the groups they belong | We mentioned how data were analysed, synthesised and reported in the results section. |
| 3. Results |  |
| PRISMA: “Excluded as did not match purpose of the review”. This statement is not simply enough, kindly elaborate for each paper the reason of exclusion. Line183: “In the final review, we included 64 studies”, yet in the PRISMA, the number of papers are 74. Descriptive characteristics of included studies | We corrected this in the PRISMA and revised it accordingly. |
| Kindly describe all papers identified by country, type of paper, author and year with citation of each paper | Revised accordingly, Table S4. |
| Move Table1 to supplementary documents | Corrected as suggested and moved in the supplementary file. |
| Line 202: Fig2 presents the summary of HSG interventions in the PHC context Is this Fig2 part of your results? In which paper did you extract this fig2? If the information of figure2 is not among the results, kindly put it in the introduction or literature review | Figure 2 is removed as all supplementary files table S3 table S4. |
| Line 164: You said that to summarize the findings you used WHO framework on governance and financing: Looking in to the summary, they are many subtitles which are not part of WHO framework such multi-sectoral engagement (line 438), community engagement (line 459),…. | These subtitles are themes under each component of the WHO framework. The WHO framework is a guiding framework in which related themes fit in each component and are explained by synthesising the findings from included studies. |
| Line 570: Our purpose of the review was to synthesize evidence rather than grade the evidence, however, the results are a simple summary of what was reported in the studies, on the contrary, the paper should synthesize the data as opposed to summarize. Results should have a synthesis and not a summary. | We agree with the editor. We synthesized the study’s findings under each component of the framework. |
| In the aim of the study and methodology, Universal health coverage appeared both in aim and search but does not come out among findings. What happened in the findings? Is it that no studies reported universal coverage? | The UHC was reported using the available data from the included studies. |
| 4. Discussion: Discuss how performing HSG (countries) are doing, what special strategies they have in terms of HSG which are making them performing and their strengths and weaknesses. Discuss countries in pools of LMICs and HICs, what are differences | We used a similar structure to the findings section. Interpretations are based on the key findings of each component of the framework. We interpreted our findings by referring to various LMICs and HICs to support the interpretation. |
| Conclusion The conclusion is not supporting the findings at all. Kindly relate your findings to the presented results | We thank the editor, and we revised the conclusion accordingly. |
| Reviewer 1 |  |
| The authors need to address inconsistencies in the document, In the abstract, the authors write that they used 64 studies in the review, but in the main document there are 74 studies. | We corrected this mistake. |
| In addition, the purpose of this scoping review is to explore the function of HSG in the context of PHC settings towards UHC but in the methodology, the research questions focused on contributions of HSG to the delivery of PHC services. The purpose fails to capture UHC which is consistently positioned as very important in the topic of HSG and PHC. | We agreed with the reviewer that we primarily focus on the contribution of UHC in the delivery of PHC, and effective delivery of PHC contributes to the UHC (directly and indirectly). We have reported and interpreted. |
| It is not clear what was done to the broad research questions, in addition it is not clear which is the broad research question and which is the final reviewed research question. See Lines 119-120 | Our main focus was to understand the contribution of HSG in delivery of PHC services to UHC. |
| Why were the six databases selected, a justification for this choice is essential. | We provided a justification for why we used six databases. |
| Line 116: The author should discuss the three concepts referred to here in this line. | We mentioned these terms |
| I suggest that authors avoid the use of disagreements by the reviewers on varying issues e.g Line 146… | We corrected it as suggested. |
| In Line 192, it is not clear how many of these studies were from, El Salvador, Ukraine, Egypt, Indonesia, and Kenya | We specified these studies. |
| In line 499, digital tools cannot form policies,  In lines 285-line 286 (who is empowered?) | We made clarification. |
| In lines 142-143. Were there specific interventions the authors were referring to?  Line 87-98 the sentence is incomplete. | We revised it and made it clear. |
