# Supplementary material for: Contribution of health system governance in delivering primary health care services for universal health coverage: A scoping review
Source: PLoS One. 2025 Feb 28;20(2):e0318244. doi: 10.1371/journal.pone.0318244 (PMC11870385; doi:10.1371/journal.pone.0318244)
Supplement: Attachment — Submitted filename: Review_contrib_Gov_PHC.docx [file pone.0318244.s008.docx]

**Review**

**Contribution of health system governance on delivery of primary health care services**

**towards universal health coverage: a scoping review**

**Introduction**

The introduction is too long and not well structured. The flow is not coming out clearly and it is difficult for the reader to follow. The topic is broad and there is a need to narrow down on the topic. Kindly narrow down and introduce the topic clearly. i.e; How HSG is very different worldwide. High income countries have a different HSG compared to Low and Middle Income Countries. Kindly make distinction between the two and briefly describe the differences between the two by focusing on one geographical area. This study used the “ Arksey and O’ Malley’s scoping review framework” methodology. Kindly describe briefly how the framework supports your methodology. The authors have given so many framework examples but are not focusing on the main framework used. Identify only 3 previous studies on the topic and briefly discuss them in the introduction. What is the research question of this study? Previous review comments on introduction have been addressed partially.

**Methodology**

Line137-138: “We identified the research question focusing on the contribution of HSG to the delivery of PHC services towards UHC”. Where is the research question??

For methodology, I have a serious problem with the search strategy. The search strategy is not clear including search terms and eligibility criteria, making it impossible to reproduce

Kindly Make two additional headings in methodology, one for “Data sources and Search strategy” and another one “inclusion and exclusion criteria”

It is also not clear how the study selection was conducted and how the synthesis of results was done. Preview comments on methodology have not been addressed

**Results**

There is a need to clarify step by step how the screening of the results was done by explaining each step how you excluded papers and reasons till 74 included papers.

The descriptive characteristics of included studies is not clear. For each paper included, attach it to a country. For example 3 papers from china, 1 paper from South Africa, …The results of this study are reported in form of summary instead of synthesis. This results provide a concise representation of main points and key findings from the literature reviewed. The study is condensing the information to highlight core aspects instead, the reporting should focus on synthesis by integrating information from different studies to generate insights, theories and understandings that goes beyond the individual contributions of papers.

It is very difficult to comment on discussion and conclusion if the methodology and results are not well done.
