# Supplementary material for: Contribution of health system governance in delivering primary health care services for universal health coverage: A scoping review
Source: PLoS One. 2025 Feb 28;20(2):e0318244. doi: 10.1371/journal.pone.0318244 (PMC11870385; doi:10.1371/journal.pone.0318244)
Supplement: Attachment — Submitted filename: Point by point response to reviewers comments.docx [file pone.0318244.s009.docx]

**Point by point to the editor's and reviewers’ comments**

Manuscript ID: PONE-D-23-27336R1

The authors’ team would like to thank you for your insightful and constructive feedback on our manuscript. We have revised it as suggested. In this document, we have responded point-by-point to your comments and clarified our concerns where necessary.

**Reviewer #1(Dr Kezia Njoroge, Senior lecturer in Public health at Liverpool John Moores University)**

**Reviewer’s comment:**  I am satisfied that the authors have addressed the raised comments. Despite the large number of comments, the authors addressed them well.

**Authors’ response: The a**uthors team would like to thank you for your time and input on our manuscript. Your comments were so insightful and constructive. We incorporated them in the revision and realise that our manuscript improved significantly.

**Reviewer #2**

**Introduction**

**Reviewer’s comment:**  The introduction is too long and not well structured. The flow is not coming out clearly and it is difficult for the reader to follow. The topic is broad and there is a need to narrow down on the topic. Kindly narrow down and introduce the topic clearly. i.e; How HSG is very different worldwide. High income countries have a different HSG compared to Low and Middle Income Countries. Kindly make distinction between the two and briefly describe the differences between the two by focusing on one geographical area.

**Authors’ response:** Thank you, reviewer, for this comment. We have revised this section. Current framing of the introduction section is the concept of health system governance, and its importance, followed by the framework of the framework in relation to primary health care and universal health coverage. Introduction of health system governance of high and low-income counties. The role of health system governance and building blocks, UHC and PHC, was introduced. Finally, the introduction section provides the rationale for the review, study objective and potential implications of the study. We believe that the current framing of the introduction section is more structured and reader-friendly.

**Reviewer’s comment:**  This study used the “ Arksey and O’ Malley’s scoping review framework” methodology. Kindly describe briefly how the framework supports your methodology. The authors have given so many framework examples but are not focusing on the main framework used. Identify only 3 previous studies on the topic and briefly discuss them in the introduction. What is the research question of this study? Previous review comments on introduction have been addressed partially.

**Authors’ response:** Again, thank you for this feedback. We removed the description of the other framework. We have focused on HSG framework only. We used “Arksey and O’ Malley’s scoping review framework” to guide our methods of scoping review, while the HSG framework is used to present our findings. In short, the former framework guides the research process while later helping to organize the findings. Study research question is included in the last paragraph of the introduction section.

**Methodology**

**Reviewer’s comment:**  Line137-138: “We identified the research question focusing on the contribution of HSG to the delivery of PHC services towards UHC”. Where is the research question??For methodology, I have a serious problem with the search strategy. The search strategy is not clear including search terms and eligibility criteria, making it impossible to reproduce. Kindly Make two additional headings in methodology, one for “Data sources and Search strategy” and another one “inclusion and exclusion criteria”
It is also not clear how the study selection was conducted and how the synthesis of results was done. Preview comments on methodology have not been addressed

**Authors’ response:** Thank you for the feedback. We have included the research question in the last paragraph of the introduction section (what are the contribution of HSG (successes and challenges) in the production and delivery of primary health care services towards UHC?). Additionally, we have added two subheadings, as suggested in the methods section.

**Results**
There is a need to clarify step by step how the screening of the results was done by explaining each step how you excluded papers and reasons till 74 included papers.
The descriptive characteristics of included studies is not clear. For each paper included, attach it to a country. For example 3 papers from china, 1 paper from South Africa, …The results of this study are reported in form of summary instead of synthesis. This results provide a concise representation of main points and key findings from the literature reviewed. The study is condensing the information to highlight core aspects instead, the reporting should focus on synthesis by integrating information from different studies to generate insights, theories and understandings that goes beyond the individual contributions of papers.

**Authors’ response:** Thank you for the feedback. We have revised it accordingly. The findings are presented based on our methods (framework-guided thematic analysis of the contents derived from studies included in the review). This analysis approach is different from what the reviewer suggested. We believe the current structure and presentation findings are useful for the readers and reader-friendly.

It is very difficult to comment on discussion and conclusion if the methodology and results are not well done.

**Authors’ response:**  We conducted this scoping review by following scoping review guidelines and analysing data using a framework guided by a thematic analysis approach. We presented findings guided by the methods (for instance, narrative explanation of themes under each component of the framework) of past published research. We framed the discussion section based on the findings and concluded based on the discussion and objective of the study. Thus, the content and organization of the discussion section are coherent with the methods and findings.

We thank reviewers for the very positive and constructive feedback on our work, and we are so grateful to the reviewers and editor.
